# Established and Emerging Roles of DEAD/H‐Box Helicases in Regulating Infection and Immunity

**DOI:** 10.1111/imr.13426

**Published:** 2024-12-02

**Authors:** Michael Parthun, Matthew E. Long, Emily A. Hemann

**Affiliations:** ^1^ Department of Microbial Infection and Immunity The Ohio State University College of Medicine Columbus Ohio USA; ^2^ Infectious Diseases Institute The Ohio State University Columbus Ohio USA; ^3^ Dorothy M. Davis Heart and Lung Research Institute The Ohio State University College of Medicine Columbus Ohio USA; ^4^ Department of Internal Medicine, Division of Pulmonary, Critical Care, and Sleep Medicine The Ohio State University College of Medicine Columbus Ohio USA

**Keywords:** DEAD‐box helicases, immunity, infection, interferon, RLRs

## Abstract

The sensing of nucleic acids by DEAD/H‐box helicases, specifically retinoic acid‐inducible gene I (RIG‐I) and melanoma differentiation‐associated protein 5 (MDA5), plays a critical role in inducing antiviral immunity following infection. However, this DEAD/H‐box helicase family includes many additional proteins whose immune functions have not been investigated. While numerous DEAD/H‐box helicases contribute to antiviral immunity, they employ diverse mechanisms beyond the direct sensing of nucleic acids. Some members have also been identified to play proviral (promoting virus replication/propagation) roles during infections, regulate other non‐viral infections, and contribute to the regulation of autoimmunity and cancer. This review synthesizes the known and emerging functions of the broader DEAD/H‐box helicase family in immune regulation and highlights ongoing efforts to target these proteins therapeutically.

## 1 Introduction

DEAD/H‐box helicases are a highly conserved family of RNA helicases named for a core Aspartate–Glutamate‐Alanine‐Aspartate/Histidine (DEAD/H) amino acid motif within their helicase domains. The members of this family generally exhibit high conservation of the helicase domain, which contains nucleic acid binding as well as ATP binding/hydrolysis domains [1, 2]. However, the specific nucleic acid moieties recognized by members of this family, and whether they are able to bind nucleic acids, vary widely, resulting in diverse functional roles [3, 4]. DEAD/H‐box helicases participate in a vast array of biological functions, including RNA processing, ribosome biogenesis, translation initiation, RNA decay, and innate immune signaling [5, 6]. Due to these essential functions, many DEAD/H‐box helicases are not only crucial factors influencing the outcome of viral infections, as they have roles in innate immune pathogen recognition pathways, but also serve as host factors exploited by viruses to promote viral replication due to their involvement in RNA processing and translation [7, 8]. Consequently, DEAD/H‐box helicases can exhibit both antiviral and proviral functions, and often the same helicase can be anti‐ or proviral depending on the specific virus and cell type involved [9]. This review will delve into the current understanding of the various functions of host DEAD/H‐box helicases in viral infections and immunity which has been an active and growing area of research as the important roles of nucleic acid sensors have discovered to contribute to a wide range of human diseases.

## 2 Antiviral Roles of DEAD/H‐Box Helicases

The DEAD/H‐box helicases most commonly associated with the restriction of viral replication are the RIG‐I‐like receptors (RLRs), which include retinoic acid‐inducible gene I (RIG‐I, gene *DDX58*), melanoma differentiation‐associated protein 5 (MDA5, gene *IFIH1*), and laboratory of genetics and physiology 2 (LGP2, gene *DHX58*) [10, 11]. The RLR subclass of DEAD/H‐box helicases is pivotal in sensing specific cytosolic nucleic acid moieties following infection or cell damage [12, 13]. RIG‐I typically binds shorter double stranded RNAs (dsRNAs) ranging from 10 to 500 base pairs that possess a 5′ terminal triphosphate or diphosphate group and lack methylation of the mRNA cap at the 2′‐O position on their 5′ end [13, 14, 15]. MDA5, on the other hand, typically binds longer dsRNAs, from 0.5 to seven kilobases, with RNA secondary structure also influencing its binding [16, 17, 18]. In contrast, rather than functioning independently LGP2 lacks a helicase domain and instead regulates RIG‐I or MDA5 to fine tune their activity through mechanisms reviewed elsewhere [19].

**FIGURE 1:**
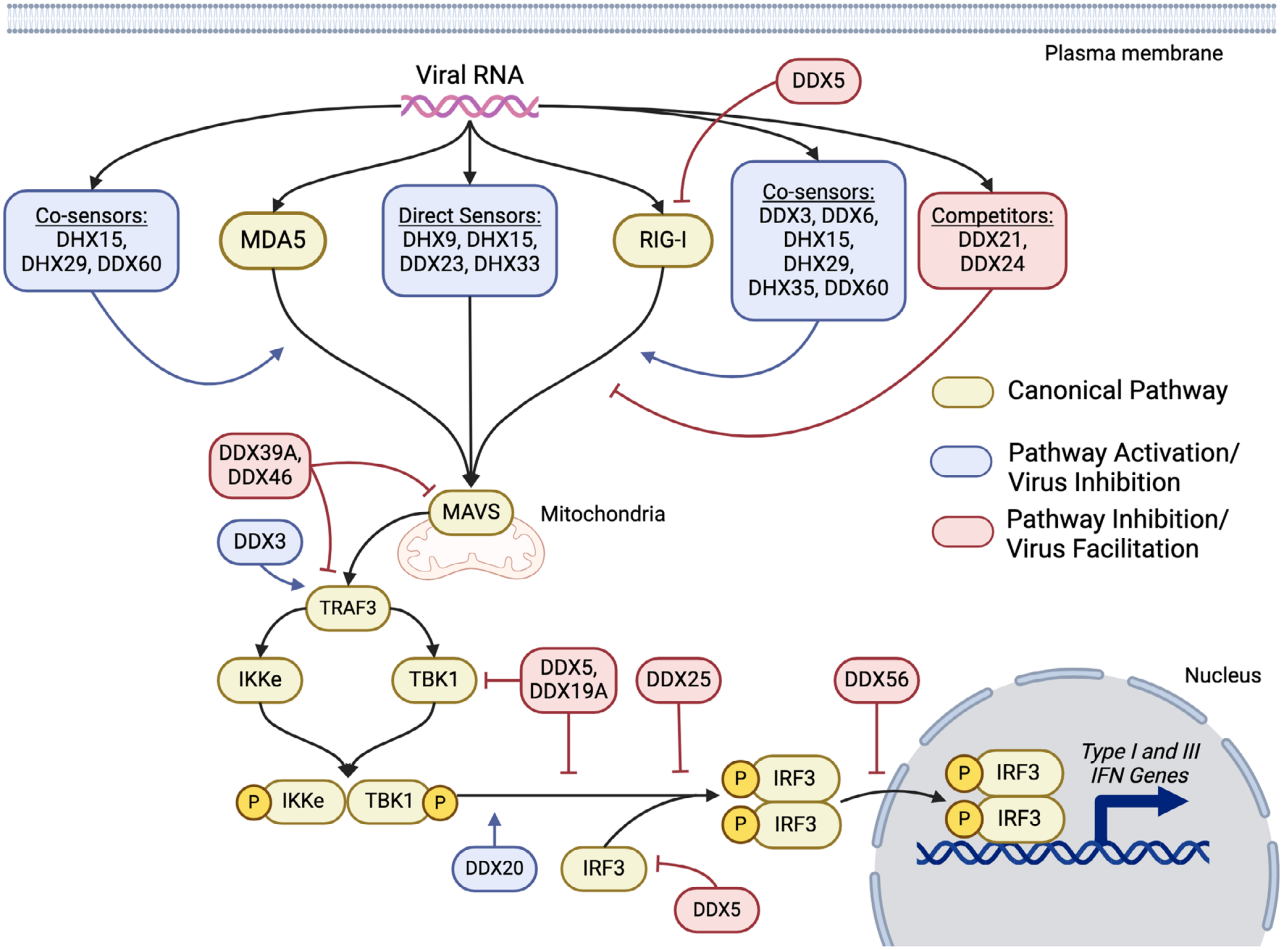
DEAD/H‐box helicases in the RLR‐IRF3 pathway during viral infection. A number of DEAD/H‐box helicases bind viral RNA leading to their activation, formation of the MAVS signalosome, downstream activation and nuclear translocation of IRF3, and subsequent expression of type I and type III IFN genes. The core canonical components of this pathway are depicted in yellow. DEAD/H‐box helicases which promote this pathway to restrict viral infection are shown in blue, and those which inhibit the activity of this pathway to facilitate viral infection are shown in red.

**FIGURE 2:**
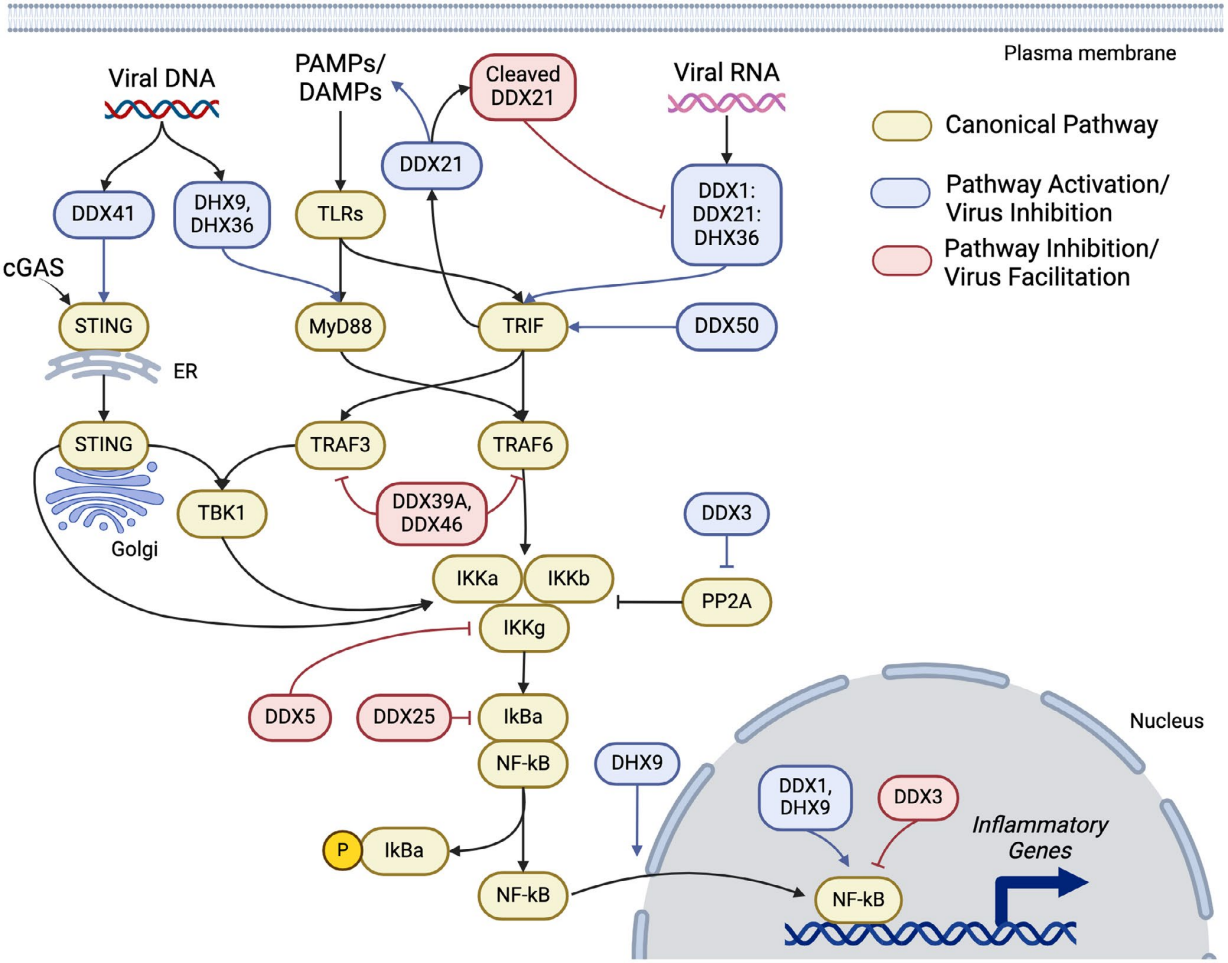
DEAD/H‐box helicases in the NF‐kB pathway during viral infection. PAMP or DAMP sensing by PRRs, including the TLRs, cGAS, or certain DEAD/H‐box helicases, causes these PRRs to complex with specific adaptors proteins, such as STING, MyD88, or TRIF. This eventually results in the degradation of the NF‐κB inhibitor IκBα and nuclear translation of NF‐κB into the nucleus, where it promotes transcription of several pro‐inflammatory gene programs. The core components of this pathway are depicted in yellow. DEAD/H‐box helicases which promote this pathway to restrict viral infection are shown in blue, and those which inhibit the activity of this pathway to facilitate viral infection are shown in red.

Upon nucleic acid binding to the RLR helicase domain, a conformational change releases the caspase activation and recruitment domain (CARD) allowing for oligomerization and transit to outer mitochondrial membranes for interaction with mitochondrial antiviral signaling protein (MAVS) via CARD‐CARD domain binding [20, 21]. This interaction leads to formation of a signalosome complex that includes RLR, MAVS, TNF receptor‐associated factor 3 (TRAF3), TANK‐binding kinase 1 (TBK1), and inhibitor of NF‐κB kinase subunit epsilon (IKKε). The activation of this complex ultimately results in the phosphorylation of interferon (IFN) regulatory factor 3 (IRF3) and IRF7 and their translocation to the nucleus to induce the transcription of type I and type III IFN and antiviral genes [14, 16, 22]. Additionally, MAVS complex formation with TRAF6 activates downstream nuclear factor kappa‐light‐chain‐enhancer of activated B cells (NF‐κB) to induce pro‐inflammatory cytokine production [23, 24]. While RLRs are the prototypical cytosolic nucleic acid sensors, several other DEAD/H‐box helicases also have integral roles in detection of cytosolic nucleic acids [7, 25] with some proteins functioning as co‐sensors with RIG‐I, while others directly sense viral nucleic acids independently of RLRs. Many of these helicases also modulate the RLR activation pathway independently of viral nucleic acid sensing, influencing downstream antiviral signaling cascades (Figures 1 and 2; Table 1).

**TABLE 1:** Roles of DEAD/H‐box helicases in infectious disease, including viral and nonviral. Functions which inhibit pathogen replication or ameliorate pathogenesis are shown in blue, and functions which facilitate pathogen replication or worsen pathogenesis are shown in red. Functions which can promote or inhibit pathogenesis depending on context are shown in black.

	Functions (Pathogen inhibition/pathogen facilitation)	References
Viruses		
DDX1	• Transcriptional coactivator of NF‐κB during TGEV infection	[26], [27], [28], [29], [30]
	• Senses IAV and Reovirus RNA to activate TRIF	
	• Regulates switch between sgmRNA and gRNA production in MHV	
	• Cofactor for HIV nuclear export complex	
DDX2	• Also known as eIF4a. Required for translation of viral proteins during many infections	[31]
DDX3	• Senses VSV RNA with RIG‐I to activate MAVS	[32], [33], [34], [35], [36], [37], [38], [39], [40], [41], [42], [43]
	• Activates TRAF3 to induce IFN‐β in SeV infection	
	• Enhances IKKε autophosphorylation to increase IRF3 phosphorylation in SeV infection	
	• Enhances PACT translation and type I interferon in response to poly(I:C), LPS, or HCV RNA	
	• Acts as a scaffold to promote NLRP3 inflammasome in BMDMs treated with LPS	
	• Component of antiviral stress granules important for control of IAV and VACV	
	• Blocks nuclear translocation and activity of NF‐κB after TNF stimulation in vitro	
	• Binds viral RNA to increase activity of the viral polymerase and enhance replication of JEV	
	• Cofactor for HIV nuclear export complex	
	• Binds VEEV and JEV RNA and enhances translation of viral proteins	
DDX4	• Increases SOCS1 degradation to enhance JAK/STAT in VSV, IAV, SeV, and HSV‐1 infection	[44]
DDX5	• Promotes readthrough of HBV polyadenylation signals leading to viral RNA decay	[45], [46], [47], [48], [49], [50]
	• Promotes TBK1 degradation to limit interferon production during SVCV infection	
	• Blocks activation of IRF3 and interferon production during VSV and HSV infection	
	• Promotes m6A addition to antiviral transcripts to degrade them and promote VSV infection	
	• Binds viral RNA to increase activity of the viral polymerase and enhance replication of JEV	
	• Assists SINV encapsidation	
DDX6	• Senses EV71 and IBV RNA with RIG‐I to activate MAVS	[51], [52], [53], [54], [55], [56]
	• Part of decapping complex which destabilizes RVFV, LACV, and ZIKV RNA	
	• Limits baseline RLR expression and activity, enhancing VEEV, DENV, VSV, and EMCV	
	• Assists Gag‐dependent HIV encapsidation	
137DHX9	• Senses reovirus and IAV to activate MAVS	[57], [58], [59], [60], [61], [62], [63], [64], [65], [66], [67], [68]
	• Binds HSV DNA to activate MyD88 and NF‐κB for IL‐6 and TNF production	
	• Transcriptional coactivator of NF‐κB during VACV infection	
	• Activates NLRP9b inflammasome after binding rotavirus RNA in intestinal epithelial cells	
	• Enhances CD8 T cell activation, differentiation and memory responses following LCMV	
	• Binds HCV, FMDV, HIV, and IAV RNA to increase viral polymerase activity and replication	
	• Binds HIV and MYXV RNA and enhances translation of viral proteins	
DHX15	• Senses SeV and VSV RNA with RIG‐I and EMCV RNA with MDA5 to activate MAVS	[69], [70], [71]
	• Senses EMCV and reovirus RNA to activate MAVS	
DDX17	• Promotes readthrough of HBV polyadenylation signals leading to viral RNA decay	[45], [72], [73], [74], [50]
	• Binds HBV and RVFV genomic RNA and blocks binding of the viral polymerase	
	• Induces expression of ZWINT during HBV infection to promote viral RNA replication	
	• Assists SINV encapsidation	
DDX19	• DDX19A binds PRRSV RNA and activates the NLRP3 inflammasome	[75], [76], [77]
	• Promotes TBK1 degradation to limit type I IFN production during EMCV infection	
	• Remodels IAV mRNPs to facilitate nuclear export	
DDX20	• Improves TBK1 and IRF3 binding to upregulate IFN‐β in VSV, HSV‐1, and SeV infection	[78]
DDX21	• Senses IAV and Reovirus RNA to activate TRIF	[26], [79], [80], [81], [82], [83], [84]
	• Increases Calgranulin B production to stimulate MyD88 pathway during IAV infection	
	• Binds to and blocks the activity of the IAV polymerase	
	• Competes with RIG‐I for SeV RNA substrates to blunt interferon production	
	• Cleaved form inhibits DDX1/DDX21/DHX36 complex during VSV and HSV infection	
	• Regulates the activity of the BDV polymerase to promote virus replication	
	• Cofactor for HIV nuclear export complex	
DDX23	• Senses VSV RNA to activate MAVS and TRIF	[85]
DDX24	• Competes with RIG‐I for VSV RNA substrates to blunt interferon production	[86], [87]
	• Assists Rev‐dependent HIV encapsidation	
DDX25	• Inhibits IRF3 and NF‐κB activation to promote infection by DENV, SeV, VSV	[88]
DHX29	• Senses SeV, IAV, and RSV‐A2 RNA with RIG‐I and EMCV RNA with MDA5 to activate MAVS	[89], [90]
DHX33	• Senses reovirus RNA to activate MAVS	[91], [92]
	• Activates NLRP3 inflammasome after binding poly(I:C) or reovirus RNA in macrophages	
DHX35	• Senses IAV and RSV‐A2 RNA with RIG‐I to activate MAVS	[93]
DHX36	• Senses IAV and NDV RNA with RIG‐I to activate PKR	[94], [26], [58]
	• Senses IAV and Reovirus RNA to activate TRIF	
	• Binds HSV DNA to activate IRF7 and produce IFN‐α in human pDCs	
DDX39A	• Retains antiviral transcripts in the nucleus to facilitate VSV infection	[95]
DDX41	• Binds STING to activate IRF3 and NF‐κB after binding HSV and *listeria m*. DNA in DCs	[96], [97]
	• Signals through STING to produce IFN‐β after binding HIV and MLV reverse transcripts	
DDX42	• Blocks the replication of many positive stranded viruses in an interferon‐independent fashion	[98]
DDX46	• Facilitates m6A removal to retain antiviral transcripts and inhibit interferon in VSV infection	[99]
DDX50	• Enhances NF‐κB and IRF3 activity to limit replication of DENV, SeV, HSV‐1, and VACV	[100], [101]
DDX56	• Binds CHIKV RNA and promotes its degradation by NMD and the RNA exosome	[102], [137], [142]
	• Blocks IRF3 nuclear translocation and interferon production during WNV and SeV infection	
	• Assists WNV encapsidation	
DDX60	• Senses VSV and SeV RNA with RIG‐I and PV RNA with MDA5 to activate MAVS	[103], [128]
	• Binds HCV and VSV RNA and enhances their degradation by the RNA exosome	
MDA5	• PRR that senses viral RNA in many different contexts to stimulate interferon production	[12]
RIG‐I	• PRR that senses viral RNA in many different contexts to stimulate interferon production	[12]
Non‐viral pathogens		
DDX3	• Enhances IFN‐β transcription independently of IRF3 during *L. monocytogenes* infection	[163], [164]
	• Shuttles *Y. enterocolitica* virulence factor out of the nucleus to limit production of IL‐10	
DDX5	• Limit basal TLR levels, IL‐6 and TNF, and pathology of *M. pneumonia*, *S. aureus* , and *P. multicoda*	[152]
DHX33	• Activates NLRP3 inflammasome after binding *E. coli* RNA in macrophages	[92]
DDX41	• Senses *L. monocytogenes* DNA and second messengers to produce STING‐dependent interferon	[96], [154], [162]
	• May sense DNA from several *Plasmodium* species and bind STING to produce interferon	
MDA5	• Senses *M. tuberculosis* RNA and induces interferon production	[153], [155], [160], [161]
	• Senses *Aspergillus fumigatus* RNA to induce interferon and protect against severe disease	
	• Reduced autophagy and increased inflammasome activation to support *M. tuberculosis*	
	• Enhances macrophage apoptosis and inhibits phagocytosis in *Candida albicans* infection	
RIG‐I	• Senses *L. monocytogenes, S. enterica*, and *M. tuberculosis* RNA to induce interferon	[156], [157], [158]

### 2.1 Viral Nucleic Acid Sensing DEAD/H‐Box Helicases

**FIGURE 3:**
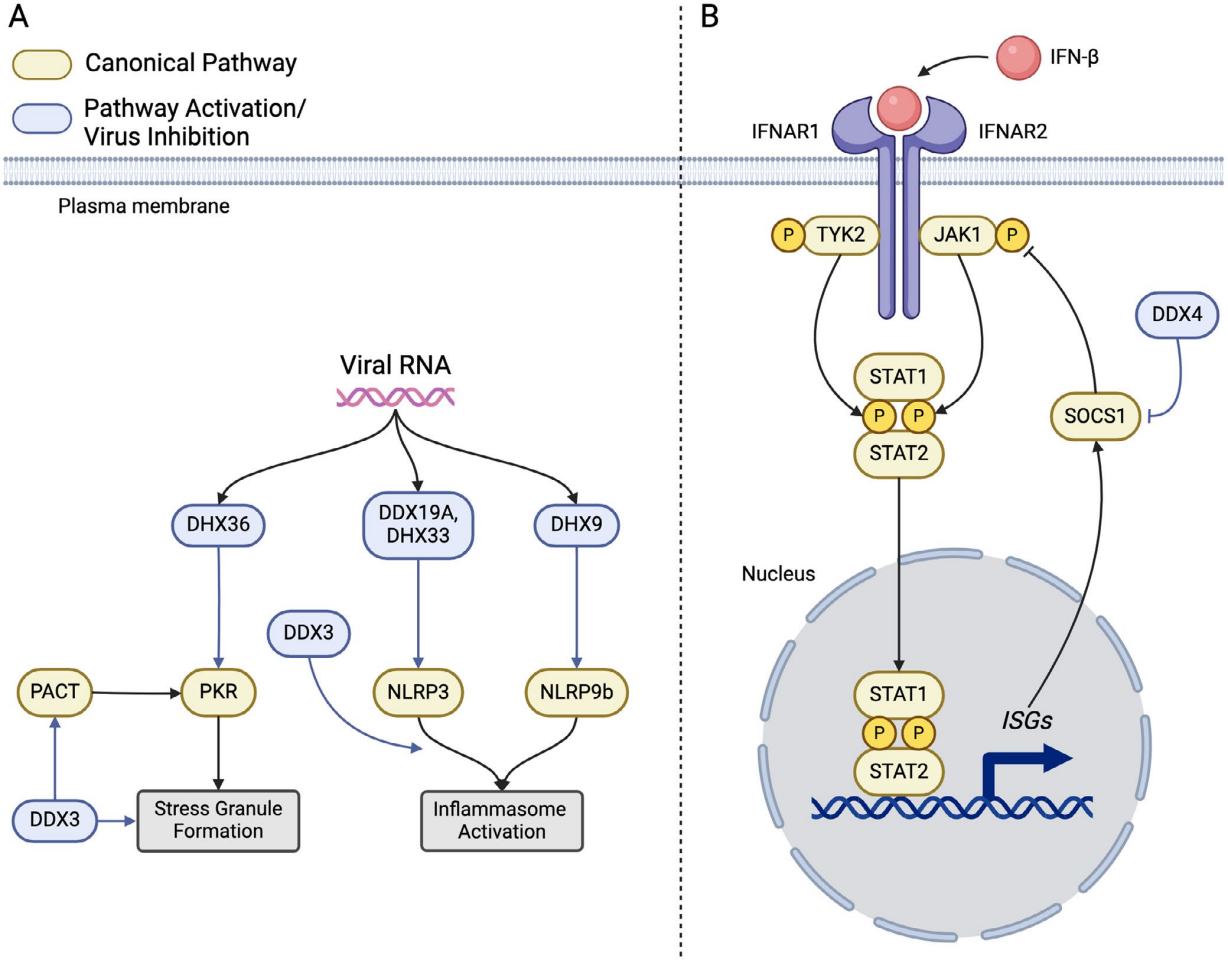
DEAD/H‐box helicases in other host inflammatory pathways during viral infection. (A) DEAD/H‐box helicases which upregulate the formation of antiviral stress granules (left) and inflammasomes (right). Several DEAD/H‐box helicases sense viral RNA to trigger the eventual formation of these structures, while DDX3 has been shown to act as a scaffold to enhance formation of both. (B) DDX4 degrades SOCS1 to remove negative feedback from JAK/STAT signaling downstream of type I IFN receptor signaling. This enhances and sustains ISG expression. The core components of each pathway are depicted in yellow. DEAD/H‐box helicases which promote each pathway to restrict viral infection are shown in blue.

Several DEAD/H‐box helicases, including DDX3, DDX6, DHX15, DHX29, DHX35, DHX36, and DDX60, bind both viral nucleic acid and canonical RLRs, augmenting the canonical antiviral signaling response [12, 32, 51, 52, 69, 89, 93, 94, 103]. DDX6 and DHX15 bind both RIG‐I via its CARD domain and viral RNA in complex with RIG‐I, which presumably strengthens RIG‐I's RNA‐binding affinity. In the case of DHX15, this interaction augments RIG‐I ATPase activity and enhances IRF3 phosphorylation and IFN induction during influenza B virus (IBV), enterovirus 71(EV71), Sendai virus (SeV), and vesicular stomatitis virus (VSV) infection [51, 52, 69]. DDX60 has also been shown to bind dsRNA and RIG‐I, augmenting type I IFN production downstream of RIG‐I during VSV and SeV infection [103]. Although they haven't been described to directly bind RNA, DDX3, DHX29, and DHX35 bind both RIG‐I as well as MAVS to enhance MAVS signaling and promote type I IFN expression in response to VSV (DDX3), SeV (DHX29), influenza A virus (IAV) (DHX29 and DHX35), and respiratory syncytial virus (RSV) (DHX29 and DHX35) [12, 32, 89]. MDA5 similarly interacts with DEAD/H‐box helicases for functional regulation. For example, DHX15, described above to interact with RIG‐I, also co‐senses RNA with MDA5, enhancing type I IFN production during encephalomyocarditis virus (EMCV) infection [69]. Although the specific mechanistic details of this interaction are less understood than those for RIG‐I and DHX15, it is plausible that DHX15 enhances binding of viral RNAs to MDA5 in a manner analogous to its interaction with RIG‐I. This ability to interact with both RLRs is not reserved solely for DHX15, as DHX29 and DDX60 also bind MDA5 and long viral dsRNA during EMCV or poliovirus infection, respectively, resulting in increased IRF3 activation and enhanced type I IFN responses [90, 103]. While these DEAD/H‐box helicases associate with RLRs in response to infection, DHX36 is unique in that it has been identified to associate with the C terminal domain (CTD) of RIG‐I in resting cells in the absence of infection. Upon infection with Newcastle disease virus (NDV) or IAV, this complex binds both viral dsRNA and the viral RNA sensor Protein Kinase R (PKR), enhancing PKRs autophosphorylation and activation. This activation of PKR leads to increased IFN production downstream of the formation of antiviral stress granules (Figure 3a, left) [94].

In contrast to the DEAD/H‐box helicases that require a RLR to sense nucleic acids, several DEAD/H‐box helicases bind viral nucleic acids leading to innate immune activation independent of association with a canonical RLR. For example, DHX9 (also known as RNA Helicase A/RHA), DHX15, DDX23, and DHX33 each independently form a signalosome with MAVS that leads to induction of antiviral and type I IFN responses following viral double stranded RNA binding. This mechanism has been observed during reovirus (DHX9, DHX15, and DHX33), IAV (DHX9), EMCV (DHX15), and VSV (DDX23) infection [57, 70, 71, 85, 91]. Notably, both DHX9 and DHX15 are capable of sensing a variety of lengths of dsDNA, 0.2–8 kilobases for DHX9 and 1.5–8 kilobases for DHX15 [51, 57]. MDA5 is also known to bind long dsRNA in a similar range, but it is currently unknown what, if any, dsRNA features might impart binding specificity for MDA5, DHX9, or DHX15 specifically. Furthermore, DHX9 has been found to complex with DHX15 in unstimulated cells and cells stimulated with poly(I:C), and the presence of either helicase was found to improve poly(I:C) binding of the other, suggesting that DHX9 and DHX15 may bind dsRNA cooperatively [51]. DHX15 also binds dsRNA cooperatively with Nod‐like receptor family pyrin domain containing 6 (NLRP6) in intestinal epithelial cells during EMCV infection, resulting in enhanced type I IFN response and reduction of disease severity [25]. This enhancement of DHX15 sensing is likely independent of the role of NLRP6 in the inflammasome, as the enhanced IFN responses and reduction in disease did not require caspase 1. While the above helicases primarily signal through MAVS, DDX23 can also signal through another adaptor protein, TIR‐domain‐containing adapter‐inducing IFN‐β (TRIF), following binding to VSV RNA, inducing NF‐κB responses alongside canonical IRF3 activation [85]. Similar signaling was also induced following direct sensing of IAV and reovirus dsRNA by a complex of DDX1, DDX21, and DHX36 [26]. Notably, DHX15 and DHX36 exhibit both RLR‐dependent and ‐independent mechanisms for direct viral nucleic acid sensing. RLRs are generally present in cells but can be upregulated as IFN‐stimulated genes (ISGs) following infection. In addition, there are multiple examples of the RLRs being targeted for degradation during infection to limit antiviral immunity [104]. Given these changes in expression of the RLRs throughout the course of infection, RLR‐dependent and independent viral sensing mechanisms may function to ensure adequate pathway activation and robust antiviral immune responses throughout infection as levels of these proteins fluctuate. A deeper understanding of these dynamics may reveal new targets for promoting antiviral responses.

While the endosomal Toll‐like receptor 9 (TLR9) is important for sensing of DNA CpG motifs for IRF3 and NF‐kB activation early upon entry, DHX9 and DHX36 are myeloid differentiation primary response 88 (MyD88)‐dependent DNA sensors localized to the cytosol, allowing them to sense viral DNA later during replication [58, 105, 106]. Specifically, DHX9 and DHX36 were found to bind CpG motifs in herpes simplex virus (HSV) DNA in human plasmacytoid dendritic cells (pDCs), leading to an NF‐κB response and an IRF7 response, respectively. Following binding of DNA to cyclic GMP‐AMP synthase (cGAS) in the cytosol, cGAS converts ATP and GTP to the second messenger cGAMP, which binds stimulator of IFN genes (STING) allowing for activation of TBK1, IRF3 and NF‐kB, similar to the mechanism utilized by the RLR pathway [107, 108, 109, 110]. However, cGAS is not the only DNA sensor which signals through STING. For example, DDX41 has also been described to signaling through STING after sensing pathogen‐associated molecular pattern (PAMP) DNA during HSV‐1 and *Listeria monocytogenes* infection in mouse dendritic cells (DCs) and THP‐1 cells, leading to activation of both IRF3 and NF‐κB [96]. In addition, during human immunodeficiency virus (HIV) and murine leukemia virus (MLV) infection, DDX41 binds to viral reverse transcripts for STING‐dependent production of IFN‐β [97]. Thus, there are multiple helicases that can initiate innate immune signaling pathways.

### 2.2 Nucleic Acid Sensing Independent Roles of DEAD/H‐Box Helicases in IFN and Antiviral Responses

While some helicases enhance antiviral responses through interactions with viral nucleic acids or direct associations with RLRs, others, like DDX3, significantly influence the pathway at multiple stages. For instance, during SeV infection, DDX3 binds to TRAF3, promoting the ubiquitination, activation, and interaction of TRAF3 with MAVS, IKKε, and IRF3 [33]. Additionally, DDX3 has been shown to interact directly with the scaffolding/dimerization domain of IKKε, enhancing autophosphorylation of IKKε, potentially by facilitating dimerization of IKKε [34]. These mechanisms highlight an important role for DDX3 in the formation and function of the MAVS signalosome and subsequent IRF3 activation and type I IFN production. In addition, DDX3 helps unwind the structured 5′ untranslated region (UTR) of Protein Activator of IFN‐Induced Protein Kinase (PACT) transcripts, improving ribosome scanning by the 40S subunit and facilitating translation of PACT in uninfected cells during basal conditions [35]. PACT binds RIG‐I and allosterically enhances its helicase activity, and can also bind to both MDA5 and dsRNA, strengthening this interaction [111, 112]. Consequently, PACT increases IFN production mediated by RIG‐I and MDA5 in response to a wide variety of viruses, including SeV, hepatitis C virus (HCV), and EMCV [113]. Notably, PACT deficiency has been identified as a significant factor contributing to the impaired type I IFN response observed in DDX3 knockdown cells exposed to poly(I:C), HCV RNA, or LPS [35].

Several additional DEAD/H‐box helicases positively regulate the IRF3 and/or NF‐κB to promote antiviral activity but through mechanisms that are independent of virus sensing. For example, DDX20 binds to both IRF3 and TBK1 as part of the MAVS signalosome and increases the affinity of the IRF3‐TBK1 interaction, promoting phosphorylation of IRF3 and antiviral activity against VSV, HSV‐1, and SeV [78]. While not known to bind viral RNA directly, DDX50 binds TRIF and enhances phosphorylation and activity of both NF‐κB and IRF3 following poly(I:C) transfection or infection with dengue virus (DENV), SeV, HSV‐1, or vaccinia virus (VACV) [100, 101]. The mechanism by which DDX50 increases activation of IRF3 and NF‐κB, and whether this effect is TRIF‐dependent, is unknown. While DDX50 has been described to act as a co‐factor for c‐Jun‐activated transcription, the localization of DDX50 remains unclear, with one study showing DDX50 in the cytoplasm functioning upstream of MAVS and another showing retainment in the cytoplasm following infection [100, 101, 114]. Similar to DDX50, DDX21 is also a nuclear‐resident DEAD/H‐box helicase, but it is able to translocate to the cytoplasm during IAV infection. Once in the cytoplasm, DDX21 mediates TRIF activation to release Calgranulin B [a host damage associated molecular pattern (DAMP)] through an unknown mechanism, which activates MyD88 and enhances NF‐kB signaling [79]. This leads to increased macrophage secretion of IL‐6 and TNF during IAV infection. In addition to its role in direct sensing of virus described above, DDX21 also plays several additional roles in regulating immunity that will be discussed later. Functioning downstream of TRIF, DDX3 has been shown to promote IKKβ activation by inhibiting the activity of the phosphatase PP2A during stimulation with poly(I:C) or TNF‐α [36]. The NF‐kB pathway is also impacted by interaction with DHX9 and DDX1. For example, DHX9 binds HSV DNA in human pDCs to facilitate nuclear translocation of NF‐kB [58]. Inside the nucleus, DDX1 and DHX9 can act as transcriptional coactivators for NF‐κB in the context of transmissible gastroenteritis virus (TGEV) and VACV infection, respectively, although viral proteins can inhibit this process [27, 28, 59, 60]. The redundancy of DEAD/H‐box helicases such as DDX1, DHX9, and DDX21 to promote NF‐kB signaling highlights their importance in establishing a robust inflammatory response to viral infections and evading viral antagonism.

Despite NF‐κB‐ and IRF3‐mediated responses critical for antiviral immune responses to control infection, cellular infection and damage can lead to programmed cell death with bidirectional consequences depending upon the context. In the canonical inflammasome pathway, Nod‐like Receptors (NLRs) act as sensors for various DAMPs and PAMPs, leading to pro‐caspase‐1 activation upon cleavage coinciding with inflammasome formation. Caspase‐1 activation can result in the cleavage of IL‐1β, IL‐18, and Gasdermin‐D into their bioactive forms, resulting in pyroptosis [115]. Several DEAD/H‐box helicases activate the inflammasome after binding to viral RNA (Figure 3a, right). DDX19A and DHX33 activate the NLRP3 inflammasome, and DHX9 activates the NLRP9b inflammasome following binding to viral RNA [61, 75, 92]. DDX3 also promotes NLRP3 inflammasome activation, although it does so independently of its nucleic acid sensing functions by acting as a scaffold to mediate its formation [37]. A recent review has more thoroughly detailed the role of DEAD/H‐box helicases in regulating cell death to control infection and disease [116]. Overall, despite their conserved structural similarity, DEAD/H‐box helicases play diverse roles in regulating multiple steps of virus sensing and signaling pathways critical for induction of inflammatory and antiviral innate immune responses. This diverse functional repertoire highlights the critical role of DEAD‐H‐box helicases beyond the RLRs in these processes.

### 2.3 Regulation of Type I IFN Signaling by DEAD/H‐Box Helicases

While DEAD/H‐box helicases play critical roles in regulating the production of IFN, several also impart control over the IFN signaling pathway itself. DDX4, for example, promotes sustained type I IFN signaling via inhibition of suppressor of cytokine signaling 1 (SOCS1), an inhibitor of Janus kinase (JAK) activity and downstream IFN signaling (Figure 3b) [117]. DDX4 was shown to block the action of ubiquitin‐specific‐processing protease 7 (USP7) on SOCS1, leading to SOCS1 degradation and restricting a wide variety of viruses including VSV, IAV, SeV, and HSV‐1 [44]. While this degradation of SOCS1 yielded an antiviral effect, whether DDX4 also contributes to pathology in vivo remains to be investigated. Further, the role of DDX4 in regulation of type III IFN responses critical for control of viral infection at mucosal barriers, and also inhibited by SOCS1, remains unknown although it is likely to be promoted by DDX4 via a similar mechanism. A more comprehensive understanding of how DDX4 and other DEAD/H‐box helicases modulate IFN responses could open new therapeutic avenues for treating various human diseases, with potential applications beyond limiting viral infection.

### 2.4 DEAD/H‐Box Helicases Regulation of Adaptive Immunity

Beyond their well‐defined roles in innate immunity, the RLRs also exert regulatory control on the development of adaptive immunity [118, 119]. For example, DCs produce IFN in an RLR‐dependent manner, which impacts antigen presentation and downstream T cell activation and function, although whether the effect on T cell activation is also due to intrinsic mechanisms in the T cell or simply a result of impaired DC function remains unknown due to a lack of model systems to address the cell‐specific roles of RLRs [120, 121, 122, 123, 124, 125]. While the specific contributions of other DEAD/H‐box helicases in adaptive immunity are less well understood, some, like DHX9, DHX36, and DDX41, have been implicated in RLR signaling and IFN production in DCs, suggesting they may also affect downstream T cell responses [58, 96]. In addition, one study has recently shown a role for DHX9 within CD8 T cells. DHX9 was found to be a crucial factor for lymphocyte‐specific protein tyrosine kinase (LCK)‐mediated zeta‐chain‐associated protein kinase 70 (ZAP70) phosphorylation following T cell receptor stimulation and is required for proper activation and differentiation of T cells [62]. Mice with T cell‐specific deficiency in DHX9 had impaired CD8 memory responses following acute LCMV infection [62]. Furthermore, in human severe acute respiratory syndrome coronavirus 2 (SARS‐CoV‐2) patients, DHX9 expression in CD8 T cells was negatively correlated with severe disease [62]. This demonstrates that DEAD/H‐box helicases could play a role in the adaptive response and is clinically relevant for human patients. However, the question of whether the RLRs, or any other DEAD/H‐box helicases, have cell‐intrinsic roles in T or B cells remains almost completely unexplored.

### 2.5 DEAD/H‐Box Helicase Interference in Viral Replication

DEAD/H‐box helicases typically exert antiviral effects by augmenting antiviral and inflammatory pathways. However, several DEAD/H‐box helicases have antiviral functions independent of inflammatory signaling. For instance, DDX3 has been shown to be crucial for the formation of antiviral stress granules, while DDX21, DDX42, and DDX60L directly bind viral RNAs or proteins to inhibit processes necessary for the viral life cycle, such as RNA replication, transcription, translation, or encapsidation [38, 80, 98, 126, 127]. On the other hand, DDX5, DDX6, DDX56, and DDX60 function by promoting the degradation of viral RNAs [45, 53, 54, 72, 73, 102, 128]. Intriguingly, DDX17 may be able to utilize multiple mechanisms to inhibit the viral life cycle and promote degradation of viral RNAs [45, 72, 73, 129].

DEAD/H‐box helicases can also inhibit viral replication by promoting viral RNA decay through several cellular mechanisms, including regulation of Nonsense‐Mediated Decay (NMD), the RNA exosome (3′–5′ decay), and decapping that leads to 5′–3′ decay [130, 131, 132]. During hepatitis B virus (HBV) infection, DDX17 and its closely related counterpart, DDX5, promote the formation of viral transcripts with abnormally long 3′ UTRs by binding to viral DNA and reducing the use of canonical transcription termination sites, possibly by altering the topology of the viral genome [45, 129]. This leads to increased decay of viral transcripts, likely through NMD. Similarly, DDX56 can bind to a conserved stem loop of Chikungunya virus (CHIKV) genomic RNA early in infection leading to viral genome NMD in a manner that was shown to be dependent upon the RNA exosome degradation complex [102]. DDX60 enhances the RNA exosome's degradation activity by unwinding and displacing proteins from HCV and VSV RNA, making them more accessible [128]. Lastly, DDX6 is known as an activator of the decapping complex [133]. While DDX6 most often binds translationally stalled host RNAs, it can also bind and destabilize a number of viral RNAs, including Rift Valley fever virus (RVFV), La Crosse encephalitis virus (LACV), and Zika virus (ZIKV) [53, 54]. Beyond limiting viral infection, cellular RNA decay mechanisms are crucial for preventing the buildup of host RNAs, which can stimulate pattern recognition receptors (PRRs) and lead to inflammation and autoimmunity [134]. Indeed, roles for DEAD/H‐box helicases in regulating cellular transcription and translation processes (including RNA decay) have been described [133]. Further understanding the role of DEAD/H‐box helicases in RNA decay in contexts beyond viral infection may lead to new targets for the treatment of such autoimmune disorders where inappropriate RNA decay contributes to disease, such as systemic lupus erythematosus (SLE), Aicardi‐Guillard syndrome (AGS), and trichohepatoenteric syndrome (THES).

Not all DEAD/H‐box helicases rely on signaling activation, cell death, or RNA decay to disrupt viral replication. Some directly bind viral RNAs or proteins and disrupt key stages in the viral life cycle. The step reported to be targeted most often is viral RNA replication. DDX17, for instance, restricts HBV and RVFV replication by binding to a pregenomic RNA stem loop, blocking the binding site of the viral polymerase [72, 73]. DDX21 also inhibits IAV by binding to and blocking the activity of the viral polymerase PB1, a mechanism that the IAV protein NS1 counters by displacing DDX21 from PB1 to restore polymerase function [80]. DDX60L is believed to use a similar sequestration mechanism to limit HCV replication, although this mechanism remains unconfirmed [127]. DDX42 blocks the replication of positive‐stranded RNA viruses, including HIV‐1, ZIKV, Japanese encephalitis virus (JEV), CHIKV, and SARS‐CoV‐2, in an IFN (IFN)‐independent manner given the inhibition of viral replication that occurred in cells lacking IRF9 and STAT1 [98]. In addition to these mechanisms, DEAD/H‐box helicases have also been described to regulate stress granule function/formation during viral infection [135]. Stress granules inhibit viral infection through mechanisms still under investigation. While there is evidence that stress granules enhance IFN signaling by concentrating key players in the RLR pathway together near viral RNAs, stress granules have also been shown to sequester viral proteins and transcripts stalling viral translation and RNA replication [38, 136]. For example, DDX3 is a crucial component of antiviral stress granules that binds to both Poly‐A‐binding protein 1 (PABP1) and eukaryotic initiation factor 4E (eIF4E), sequestering them in stress granules and blocking translation in response to stress stimuli including sodium arsenite, heat shock, or dithiothreitol [126]. Knockdown or inhibition of DDX3 prevented the formation of stress granules in response to IAV infection, thereby enhancing virus replication [38]. Interestingly, both IAV and VACV encode viral proteins (NS1 and K7, respectively) that inhibit DDX3‐mediated stress granule formation by blocking its interactions with PABP1 and eIF4E [38, 39]. These findings highlight the significance of DDX3 in formation of stress granules, and underscores the importance of further understanding the mechanisms by which stress granules function and their role in antiviral defense.

## 3 Proviral Roles of DEAD/H‐Box Helicases

### 3.1 Inhibition of Host Immune Responses by DEAD/H‐Box Helicases

While many DEAD/H‐box helicases have antiviral functions, some actually promote viral replication by inhibiting host immune responses. DEAD/H‐box helicases negatively regulate multiple steps of the virus sensing and antiviral signaling pathways already discussed [7, 25, 26, 40, 46, 47, 48, 55, 76, 81, 86, 88, 95, 99, 137]. Generally, it is thought that this downregulation of antiviral signaling pathways is an attempt to prevent excessive inflammation [55]. However, premature dampening of inflammation can prevent the host from efficiently controlling virus infection, leading to prolonged replication and tissue damage [76, 81, 86]. As an example, both DDX24 and DDX21 compete with RIG‐I for dsRNA substrates during VSV and SeV infection, respectively [81, 86]. This competition sequesters dsRNA away from RIG‐I and inhibits the IFN response. Additionally, DDX24 forms a complex with FAS‐associated death domain protein (FADD) and receptor interacting serine–threonine protein kinase 1 (RIPK1), two proteins associated with the MAVS signalosome that enhance IRF3 and NF‐κB activity downstream of RIG‐I [86, 138, 139]. This sequestration was shown to block interaction between RIPK1 and IRF7, further inhibiting IFN production during SeV infection.

As already described above, DDX21 has been shown to form a complex with DDX1 and DHX36, allowing for dsRNA binding and activation of IRF3 and NF‐κB [26]. However, DDX21 can undergo caspase‐dependent cleavage during VSV and HSV infection, creating a non‐functional fragment that competes with full length, functional DDX21 for formation of this DDX1/DDX21/DHX36 complex, inhibiting downstream IFN responses [82]. The net impact of DDX21 during infection likely depends on virus‐ and cell‐type‐specific factors such as the activation of caspases and the expression of other helicases like RIG‐I, DDX1, and DHX36. A better understanding of DDX21 activity in these contexts could provide insight into mechanisms that tune the innate immune response. DDX6 also limits RLR sensing of host RNAs. In resting cells, DDX6 promotes the uncapping and degradation of host mRNAs to prevent their recognition by RLRs, limiting baseline IFN production and ISG expression [55]. This is beneficial when no infection is present as it prevents unnecessary and potentially deleterious inflammation. However, knockdown of DDX6 induced expression of ISGs and led to an antiviral state that inhibited cellular infection with Venezuelan equine encephalitis virus (VEEV), DENV, VSV, and EMCV [55]. While they must be carefully regulated to avoid detrimental inflammation, development of strategies to modulate DDX6 expression may serve to prime cells to mount a rapid antiviral response in high risk or viral exposure situations to potentially limit infection and/or disease.

Several DEAD/H‐box helicases have been demonstrated to inhibit IRF3 activation by multiple different mechanisms. For example, DDX5 and DDX19A promote TBK1 degradation, preventing the interaction of TBK1 with IRF3 and resulting in decreased IRF3 activation and IFN production during spring viremia of carp virus (SVCV) and EMCV infection, respectively [46, 76]. While the mechanism is not known, DDX25 also reduces phosphorylation and activation of IRF3 in addition to inhibiting degradation of NF‐κB inhibitor alpha (IκBα) and reducing subsequent NF‐κB activity during DENV, SeV, and VSV infection [88]. DDX5 has also been shown to enhance dephosphorylation of IRF3, reducing its activity during VSV and HSV infections [47]. During West Nile virus (WNV) and SeV infection DDX56 blocks interaction of nuclear transport protein importin‐5 (IPO5) with IRF3 and prevents its nuclear localization for activation of antiviral gene programs [137]. DDX3 also inhibits inflammation by binding to p65 and preventing nuclear translocation of NF‐κB following stimulation with TNF [40]. However, this role for DDX3 has yet to be confirmed in the context of infection. Clearly, stringent regulation of the IRF3 and NF‐κB pathways is critical to prevent tissue damage during infection as evidenced by the orthogonal mechanisms multiple DEAD/H‐box helicases utilize to finely tune the activation and function of these transcription factors.

A number of DEAD/H‐box helicases decrease the stability and limit translation of host transcripts, indirectly dampening antiviral responses to promote viral replication. For example, DDX39A has been described to retain key antiviral transcripts such as MAVS, TRAF3, and TRAF6 in the nucleus, inhibiting their translation leading to increased VSV replication [95, 99]. DDX46 recruits the N6‐methyladenosine (m6A) eraser protein AlkB homolog 5 (ALKBH5) to remove m6A modifications from a number of host mRNA transcripts, including MAVS, TRAF3, and TRAF6, sequestering them in the nucleus and inhibiting IFN responses during VSV [95, 99]. Conversely, DDX5 binds antiviral transcripts such as NF‐κB p65 and IKKγ and recruits the m6A writer protein methyltransferase‐like 3 (METTL3) to stimulate the addition of m6A modifications [48]. Once in the cytosol these transcripts were bound by YTH domain family 2 (YTHDF2), an m6A reader protein known to induce RNA degradation, resulting in reduced inflammation and increased titers during VSV infection [48, 140]. DDX39A, DDX5, and DDX46 all bind far more host mRNAs than just these key antiviral transcripts and have known effects on nuclear export (DDX39A, DDX46) and m6A modification (DDX5, DDX46) [48, 95, 99]. Thus, it is likely that the role these helicases play in viral infection is far broader and more nuanced than is currently understood.

In summary, many DEAD/H‐box helicases, and particularly those that have been more comprehensively investigated, have both antiviral and proviral effects depending on the virus and cellular context. This emerging concept complicates the potential role of targeting these molecules for therapeutics as they have largely been shown to function in both pro‐ and antiviral fashion depending upon the context. Thus, future studies to understand the cell‐ and virus‐specific factors which cause these helicases to have net pro‐ or antiviral effects are needed.

### 3.2 Facilitation of Viral Replication by DEAD/H‐Box Helicases

As DEAD/H‐box helicases play critical roles in host RNA processing machinery, they are often exploited by viruses to abet multiple steps in the viral life cycle [1, 25]. Several helicases, including DDX1, DDX3, DDX5, DHX9, DDX17, and DDX21 assist in the transcription and replication of viral RNAs [29, 41, 49, 63, 64, 65, 66, 74, 83, 141]. Once viral mRNAs are produced, they must be exported from the nucleus for splicing and translation, a process which is facilitated by DDX1, DDX3, DDX19, and DDX21 [30, 42, 77, 84]. When viral mRNAs are ready for translation, DDX2, DDX3, and DHX9 are exploited to assist in this process [31, 41, 43, 67, 68]. Lastly, DDX5, DDX6, DDX17, DDX24, and DDX56 all enhance the packaging of viral genomes into capsids and the production of new infectious virions [50, 56, 87, 142, 143]. Given the role of DEAD/H‐box proteins as RNA helicases, it is unsurprising that proviral DEAD/H‐box helicases most commonly enhance the production of viral RNAs. Viruses generate RNA for several purposes: as replication intermediates, mRNA for protein synthesis, and genomic RNA (gRNA) to be packaged into new virions [144, 145]. Viruses promote these crucial functions by coopting many different DEAD/H‐box helicases to enhance the activity of the viral polymerase through a variety of mechanisms [29, 41, 49, 63, 64, 65, 66, 74, 83, 141].

The most common mechanism by which DEAD/H‐box helicases promote the production of viral RNA that has been described is through the removal of bound proteins from untranslated regions (UTRs) and thereby improving access for the viral polymerase. DHX9 exemplifies this mechanism by promoting viral RNA replication and transcription across several viruses including HCV, foot‐and‐mouth disease virus (FMDV), HIV, and IAV [63, 64, 65, 66, 141]. Studies have shown DHX9 binds both the viral polymerase and the 5′ UTR of viral gRNA, enhancing the polymerase activity to promote viral replication. However, the specific alterations that DHX9 induces in 5′ UTR secondary structures vary across viruses. For example, DHX9 was shown to promote looping of viral RNA during HCV infection and was shown to unwind G‐quadruplexes and remove bound proteins on viral RNA during HIV infection, but the mechanism by which DHX9 may modulate the secondary structure of IAV and FMDV viral RNA is unknown [63, 66, 141]. Similar roles have been observed for DDX3 and DDX5, which were found to bind the JEV viral polymerase alongside the 5′ and 3′ UTRs of JEV gRNAs, presumably assisting viral polymerase activity by unwinding RNA duplexes created as replication intermediates [41, 49].

Other DEAD/H‐box helicases, such as DDX1 and DDX21, do not strictly promote viral RNA production. Instead, these helicases regulate the activity of viral polymerase and control the viral life cycle [29, 83]. For example, in the case of mouse hepatitis virus (MHV), DDX1 helps balance the production of subgenomic mRNAs (sgmRNAs), which are translated into viral proteins, and full‐length genomic RNAs, which are needed for new virions [146, 147]. DDX1 binds to MHV gRNA and promotes readthrough of transcription‐regulating sequences (TRSs), favoring the production of gRNAs over sgmRNAs [29]. Intriguingly, this effect is reminiscent of a previously discussed mechanism in which DDX5 and DDX17 promote readthrough of stop signals in the HBV genome [45]. However, in that case, readthrough leads to viral RNAs degradation by NMD, resulting in an antiviral effect. Thus, it seems that the function of promoting readthrough on viral RNAs is conserved among some members of the DEAD/H‐box helicase family, with opposing effects on viral replication based on the specific context. DDX21 also helps tune the activity of the viral polymerase, as it was reported that during Borna disease virus (BDV) infection DDX21 participates in a negative feedback loop that limits polymerase activity to avoid host cell detection [83]. DDX21 binds the 5′ UTR of the BDV X protein, inhibiting its translation and increasing nuclear levels of the BDV P protein, a polymerase cofactor [83]. However, P protein can also bind DDX21 and prevent it from downregulating the X protein. This forms a negative feedback loop that keeps nuclear levels of P protein in check and controls viral polymerase activity. Although this mechanism may have initially evolved as an antiviral response, it has been co‐opted by the virus to regulate its own life cycle. DDX17 has also been shown to promote production of viral RNAs, although the mechanism is still emerging. DDX17 deficiency was found to restrict replication of HBV in mice and block production of HBV RNA and DNA replication intermediates in HepG2‐NTCP cells [74]. Interestingly, DDX17 appears to promote viral replication by inducing the expression of ZW10 Interacting Kinetochore Protein (ZWINT), a kinetochore protein with no previously understood role in viral replication [148]. While the exact mechanism remains unclear, overexpression of ZWINT rescues the replication defect in DDX17‐deficient cells, indicating a potential role for ZWINT in the DDX17‐mediated enhancement of HBV RNA replication [74].

Further, during HIV infection, DDX1, DDX3, and DDX21 have been described to act as cofactors for Rev., an HIV protein that facilitates the nuclear export of viral mRNAs for the purpose of splicing and translation [30, 42, 84, 149, 150]. These helicases bind both Rev. and Rev‐responsive elements on viral mRNA, aiding the formation of Rev. nuclear export complexes. The life cycle of HIV is extremely well studied, and as such multiple DEAD/H‐box helicases which facilitate this process have been identified [50, 68, 142]. However, other viruses have also coopted DEAD/H‐box helicases to assist with nuclear export. For instance, DDX19 facilitates the export of viral messenger ribonucleoprotein complexes (mRNPs) during IAV infection, and other viruses like HBV, human papilloma virus (HPV), and HSV‐1 also rely on nuclear export of viral mRNAs at some point in their life cycles [77, 151].

The host translation complex includes several DEAD/H‐box helicases as crucial components, and because of this, some helicases have been coopted by a variety of viruses to translate viral proteins. For example, DDX2, more commonly known as eukaryotic initiation factor 4A (eIF4A), has a prominent role in facilitating these mechanisms. DDX2/eIF4A is a core component of the translation initiation complex and is responsible for unwinding the 5′ UTR of mRNAs and recruiting them to the ribosome. As such, DDX2/eIF4A is required for most viral translation except for internal ribosome entry site (IRES)‐mediated translation. This broad regulation of translation has made DDX2/eIF4A inhibitors attractive targets for the development of broad‐spectrum antivirals [31]. Other helicases, such as DDX3 and DHX9, also assist in viral mRNA translation by unwinding 5′ UTRs and facilitating ribosome recruitment, although their roles are more specific to certain viruses. DDX3 plays an important role in the translation of VEEV and JEV viral proteins, [41, 43] while DHX9 is critical for translation of HIV and myxoma virus (MYXV) proteins [67, 68].

The final step of the viral life cycle is the production and release of new infectious virions from the host cell. For viruses with a capsid, this involves encapsidation, the assembly of capsid proteins around a viral genome. Viruses often exploit DEAD/H‐box helicases during this step by using them to promote association of the capsid proteins and viral gRNA. This mechanism is observed across several viruses. DDX56 is coopted by WNV, and DDX5 and DDX17 are coopted by SINV for encapsidation [50, 142]. HIV exploits both DDX6 and DDX24, although through different pathways. DDX6 is involved in the Gag‐dependent encapsidation pathway, while DDX24 participates in the Rev‐dependent pathway [56, 87]. Other viruses, like porcine circovirus type 2 (PCV2), may also use helicases during this process, as DDX21 has been found to bind its capsid protein, though the exact role remains unclear [143]. Given the variety of helicases involved in different viral life cycle stages, different viruses likely utilize different DEAD/H‐box helicases to assist with this process. As CRISPR technologies become more widely available, high‐throughput knockout screens of DEAD/H‐box helicases may help identify those that are essential for different viruses with varying viral life cycles.

## 4 DEAD/H‐Box Helicases Beyond Viral Infections

### 4.1 Roles in Nonviral Infections

While DEAD/H‐box helicases are best known for their roles in viral infections, it is increasingly recognized that these proteins also play important roles in other nonviral infections [116]. Infection by these nonviral pathogens is enhanced by the negative regulation of PAMP sensing and inflammatory pathways by a number of DEAD/H‐box helicases, including DDX5 and MDA5 [152, 153]. Other DEAD/H‐box helicases restrict infection by these pathogens, usually by sensing nucleic acid‐based PAMPs produced during infection [92, 96, 154, 155]. Among nonviral pathogens, intracellular bacteria are the most common targets of DEAD/H‐box helicases, likely due to their cytoplasmic replication, which allows bacterial PAMPs to interact with cytosolic helicases, including bacterial RNA, DNA, and nucleotide‐based second messengers such as c‐di‐GMP and c‐di‐AMP. For instance, bacterial RNA can activate RIG‐I to induce IFN expression during infection by several bacterial species, including *Listeria monocytogenes, Salmonella enterica, and Mycobacterium tuberculosis* [156, 157, 158, 159]. While MDA5 is also capable of sensing bacterial RNA, particularly during *M. tuberculosis* , the downstream mechanism by which it influences bacterial replication is still being elucidated [160, 161]. In undifferentiated monocytic THP‐1 cells, MDA5 knockdown was found to increase *M. tuberculosis* infection and replication in THP‐1 cells [160]. However, a separate study found that when differentiated THP‐1 cells were treated with IFN‐γ to more closely resemble alveolar macrophages, MDA5 knockdown instead decreased *M. tuberculosis* infection [161]. Interestingly, IFN‐β production was not dependent upon MDA5 in the latter case. Rather, MDA5 was described to enhance inflammasome activity and reduce autophagy to facilitate *M. tuberculosis* survival. Together, these studies indicate that the role of MDA5 in bacterial infection may be more complex than nucleic acid sensing for induction of IFN and depend upon cell type and immune cell activation status.

Besides the RLRs, DDX41 and DHX33 have also been reported to sense bacterial nucleic acids. Although DDX41 has been well described as a viral DNA sensor, it also senses bacterial DNA and the second messengers c‐di‐GMP and c‐di‐AMP [96, 154]. Mechanistically, after binding these PAMPs, DDX41 is activated by Burton's tyrosine kinase (BTK), which also facilitates binding to STING to induce IFN during *L. monocytogenes* infection of dendritic cells [162]. DHX33 was also found to bind directly to transfected *Escherichia coli* mRNA, leading to NLRP3 inflammasome activation, suggesting this mechanism may be relevant to infection with intracellular bacteria, but this remains to be investigated [92]. While they have not been investigated, it is likely that other DEAD/H‐box helicases have similar functions. For example, DHX9 and DHX36, which are already known to bind DNA, are especially likely to act as bacterial sensors [58].

Some helicases do not directly sense bacterial PAMPs but instead regulate the host's response to bacterial infections by modulating host or bacterial proteins. For example, during *L. monocytogenes* infection, DDX3 becomes phosphorylated by TBK1 and associates with the IFN‐β promoter, enhancing IFN‐β transcription independently of IRF3 [163]. However, whether DDX3 functions as an independent transcription factor or enhances the activity of another transcription factor like NF‐κB, similar to DDX1 and DHX9, has not yet been determined [27, 60]. DDX3 has also been described to bind the *Yersinia enterocolitica* virulence factor YopM in the nucleus of macrophages during infection. This is required for shuttling YopM out of the nucleus, and limits transcription of the immunosuppressive cytokine *IL10*, that would be beneficial for bacterial replication in the macrophage [164]. Whether DDX3 can modulate the activity of other bacterial proteins is unknown, but this role for DEAD/H‐box helicases in nuclear export has been described for viral products as well. While DDX3 indirectly modulates the host response to limit bacterial infection, DDX5 modulates host immunity in a fashion that must be fine‐tuned to allow for control of infection while preventing aberrant inflammation‐mediated tissue damage. During homeostasis, DDX5 complexes with the proteins METTL3 and METTL14 to enhance the writing of m6A modifications on a number of transcripts, including the antibacterial PRRs *Tlr2* and *Tlr4*. This leads to their degradation and reduced protein levels of these critical sensors, limiting potential inappropriate immune activation [152]. During infection with *Mycoplasma pneumonia*, *Staphylococcus aureus* , and *Pasteurella multicoda* DDX5 is degraded, preventing decay of *Tlr2* and *Tlr4* transcripts and promoting upregulated protein levels of TLR2 and TLR4 critical for induction of antibacterial responses [152]. Thus, DDX5 contributes to an important positive feedback loop regulating TLR‐mediated inflammation. This mechanism demonstrates the potential importance of DEAD/H‐box helicase‐mediated balance of inflammatory responses.

The potential roles of DEAD/H‐box helicases in fungal infection are currently less well understood, with limited studies detailing their potential functions. However, both pro‐ and anti‐fungal roles have been identified for MDA5, suggesting that these pathways are also likely relevant for host‐pathogen interactions with fungi. For example, MDA5 was found to sense fungal dsRNA in the cytosol of macrophages during infection by *Aspergillus fumigatus* in mice, resulting in a protective MAVS‐mediated type I IFN response [155]. However, during *Candida albicans* infection MDA5 was recently shown to exacerbate disease and reduce survival of mice, with *Ifih1* knockout (MDA5 gene) animals displaying significant protection [153]. Intriguingly, MDA5 did not mediate susceptibility through enhancement of levels of type I or type III IFN during *C. albicans* infection; these were equivalent in WT and MDA5 knockout. Instead, MDA5 was found to promote cellular macrophage apoptosis and inhibit the phagocytosis and killing functions of macrophages that are critical for control of infection. Clearly, the role of MDA5 in fungal infections is complex, and its net impact may depend on pathogen‐, cell‐, and tissue‐specific factors that have yet to be identified.

Very little is known regarding the role of DEAD/H‐box helicases in parasitic infections; however, one study has identified DDX41 as a potential sensor of DNA from *Plasmodium* parasites, the causative agent of malaria, in a manner dependent on activation by BTK described above [162]. Infection of BTK knockout mice with *Plasmodium yoelii* led to higher parasitemia and lower survival as compared to WT mice, suggesting activation of DDX41 may be crucial for sensing of *Plasmodium* DNA. However, it is still possible that BTK is exerting this antiparasitic effect through some mechanism besides DDX41. Further investigating the role of DDX41 specifically in regulation of *Plasmodium* infection would confirm DDX41 as a novel sensor of *Plasmodium* DNA and definitively identify DDX41 as an antimalarial factor. In summary, while much of the research on DEAD/H‐box helicases has focused on their roles in viral infections, these helicases are also involved in a wide range of nonviral infections and likely serve at the interface of host‐pathogen interactions for all microbes. Further exploration into their diverse functions could lead to the development of new therapeutic strategies for bacterial, fungal, and parasitic infections.

## 5 DEAD/H‐Box Helicases in Non‐Infectious Disease

While DEAD/H‐box helicases have been extensively studied in the context of infections, they also play critical roles in various other human diseases. Notably, several autoimmune diseases are believed to stem, at least in part, from dysregulation of the RLRs [165]. The importance of the RLRs in inducing IFN responses means that their dysregulation can lead to excessive inflammation and autoimmunity, such as in type I diabetes (T1D), systemic lupus erythematosus (SLE), and Singleton‐Merten syndrome (SMS) and Aicardi‐Goutieres Syndrome (AGS) [166, 167, 168, 169, 170, 171, 172, 173, 174, 175, 176, 177, 178]. Moreover, given their involvement in DNA damage repair, RNA processing, and cell cycle regulation, DEAD/H‐box helicases are key players in the context of cancer, where they regulate both oncogenes and tumor suppressor genes [179, 180]. As such, DEAD/H‐box helicases have both oncogenic and tumor‐suppressing functions in different cancers and in different contexts. Below, we will briefly review the known roles of DEAD/H‐box helicases in autoimmunity and cancer (Table 2).

**TABLE 2:** Roles of DEAD/H‐box helicases in non‐infectious diseases, including autoimmunity and cancer.

	Functions	References
Autoimmunity		
DDX39B	• Required for splicing and expression of FOXP3, making it important for Treg development	[189]
DDX41	• May sense R loop buildup during AGS and produce interferon to contribute to pathology	[186], [188]
MDA5	• Gain‐of function mutations are associated with AGS, SMS and SLE in humans	[167], [168], [169], [170], [174], [175], [176], [177], [178]
	• Gain‐of function mutations result in spontaneous SLE in mice	
	• Gain‐of function mutations are associated with T1D in humans and mice	
RIG‐I	• Gain‐of function mutations are associated with SLE and SMS in humans	[167], [171], [172], [173], [181], [182]
	• Psoriasis patients have high RIG‐I levels in keratinocytes	
	• Rheumatoid arthritis patients have high RIG‐I levels in synovial cells	
Cancer		
DDX3	• High expression is correlated with HCC, colorectal cancer, and lung cancer	[192], [193], [194], [200], [201]
	• Activates β‐catenin to promote proliferation in colorectal cancer and lung cancer	
	• Upregulates expression of tumor suppressor p21 in HBV‐related HCC and HPV‐related lung cancer	
DDX5	• Promotes proliferation and tumorigenesis in lung cancer and colorectal cancer by activating β‐catenin	[216], [217], [218]
	• Acts as a transcriptional coactivator of androgen receptor to promote prostate cancer malignancy	
DHX9	• Associates with BRCA1 for recruitment to RNA Pol II complex for DNA damage repair	[206], [207], [208], [209], [210], [211]
	• SNPs associated with breast cancer risk	
	• High expression has been correlated with prostate cancer and lung cancer in humans	
	• Binds the promoter of and upregulates tumor suppressor p16INK4a	

### 5.1 Autoimmunity and DEAD/H‐Box Helicases

Most research linking DEAD/H‐box helicases and autoimmunity has largely focused on the RLRs, specifically RIG‐I and MDA5. As described above, these receptors are key inducers of the IFN pathway, and mutations affecting their activity can have profound effects on the development of autoimmune disease [165]. In general, gain‐of‐function mutations of RIG‐I and MDA5 produce uncontrolled inflammation through multiple mechanisms [170]. This uncontrolled inflammation can contribute to a variety of autoimmune diseases, including SLE, T1D, rheumatoid arthritis (RA), psoriasis, SMS, and AGS [166, 167, 168, 169, 170, 171, 172, 173, 174, 175, 176, 177, 178, 181, 182]. SLE is a complex autoimmune disorder characterized by the presence of autoantibodies against nucleic acids and is strongly associated with aberrant type 1 IFN expression [166]. Gain‐of‐function mutations of both RIG‐I and MDA5 were associated with increased SLE disease severity in human patients [166, 167]. Additionally, induced gain‐of‐function mutations of MDA5 resulted in spontaneous development of SLE in murine models [168, 169, 170]. Similarly, SMS, a rare disorder marked by aortic calcification, glaucoma, and skeletal dysplasia, is also associated with gain‐of‐function mutations in RIG‐I and MDA5 [171, 172, 173, 174]. Gain‐of‐function mutations in MDA5 have also been associated with T1D in humans and have been shown to cause spontaneous diabetes in mice [175]. In T1D, gain‐of‐function mutations in MDA5 lead to inappropriate sterile inflammation that likely promotes self‐reactive T cells and subsequent T cell‐mediated killing of islet cells. Conversely, loss‐of‐function mutations that inhibit the activity of MDA5 can protect against the development of T1D in murine models and are associated with a lower incidence of T1D in humans [176, 177]. No polymorphisms in RIG‐I or MDA5 have been associated with psoriasis or RA. However, high levels of RIG‐I protein have been identified in keratinocytes and synovial cells from psoriasis and RA patients, respectively, indicating that RIG‐I may be involved in the pathologies of these inflammatory conditions [181, 182].

The autoimmune disease most associated with DEAD/H‐box helicases is AGS, a neurological autoimmune disorder characterized by type 1 IFNopathy triggered by host nucleic acids [183, 184]. As such, it is associated with mutations in a number of proteins involved in nucleic acid degradation and repair, including three‐prime repair exonuclease 1 (TREX1), RNASEH2, and RNA‐specific adenosine deaminase 1 (ADAR) [185]. AGS is also associated with gain‐of‐function mutations in MDA5, which cause IFN overproduction and chronic inflammation [178]. Additionally, Aicardi‐Goutieres Syndrome (AGS), a neurological autoimmune disorder, is thought to be caused in some cases by accumulation of R loops, DNA–RNA hybrid structures that contribute to cellular stress [186]. Several DEAD/H‐box helicases are involved in the formation and resolution of R loops, with DDX41 specifically contributing to the production of IFN in response to R loops, suggesting a role for these helicases in the etiology of AGS [187, 188]. However, mechanistic confirmation of these links between R loops, non‐RLR DEAD/H‐box helicases, and AGS remains to be described. Although much of the focus has been on RLRs, other DEAD/H‐box helicases may also have unexplored roles in autoimmunity. For example, DDX39B is required for the proper splicing and expression of forkhead box P3 (FOXP3), a transcription factor crucial for development of Tregs [189]. Given that Tregs help limit T‐cell‐mediated autoimmunity, DDX39B may be important in preventing the development of a number of autoimmune disorders [190, 191]. It is likely that there are further undiscovered connections between the rest of the DEAD/H‐box helicase family and autoimmune disorders, given the importance of these helicases in regulating several inflammatory pathways. Studies investigating knockouts of other members of the DEAD/H‐box helicase family in the context of diseases such as SLE, T1D, and AGS may identify new mechanisms for the pathology of such diseases and open new avenues for the development of treatments for these debilitating conditions.

### 5.2 DEAD/H‐Box Helicases in Cancer

While a very large number of DEAD/H‐box helicases have been associated with cancer development or resistance through large genome‐wide association study (GWAS), a select number of them have been extensively studied to determine their roles in specific cancers [179]. A number of recent reviews have covered this topic in depth, so here we will only briefly summarize the current major findings in the field [179, 180].

DDX3, DDX5, and DHX9 are among the DEAD/H‐box helicases which have been most closely linked to cancer [180]. DDX3 is a putative oncogene, as it is detected at high levels in a number of different tumors, including hepatocellular carcinoma (HCC), colorectal cancer, lung cancer, and many others [180, 192, 193, 194]. This may be due to its ability to promote translation of cyclin E1 and thereby promote cell growth [195]. It also has been implicated in preventing TNF‐related apoptosis‐inducing ligand 2 (TRAIL2)‐mediated apoptosis, as well as promoting a number of other pathways involved in cancer pathology, including the Wnt, Snail, and hypoxia‐inducible factor 1‐alpha (HIF‐1a) pathways [196, 197, 198, 199]. However, DDX3 has also been implicated as a tumor suppressor, especially in cancers associated with viral infections such as HBV‐related HCC and HPV‐related lung cancer [200, 201]. This may be due to its previously described ability to restrict viral infection, although it has also been shown to suppress tumor growth in cancers unrelated to viral infection such as colorectal cancer and breast cancer [202, 203]. In sum, these studies indicate that the effect of DDX3 on cancer progression is complex and context dependent, similar to its effects on viral infection. DDX3 has been a very popular target for the development of anticancer drugs with a number of small molecule inhibitors of DDX3, including RK‐33, having been developed. RK‐33 shows potent antiproliferative effects and sensitization of cancer cells to radiation at low concentrations in animal systems and human cancer cells in vitro [204].

DHX9 can also have varied oncogenic and tumor suppressor activities due to its ability to regulate transcription of a number of oncogenes and tumor suppressors, as well as modulate the activity of the resulting proteins [205]. For example, DHX9 has been shown to facilitate interaction of breast cancer type 1 susceptibility protein (BRCA1), a DNA damage repair protein, with the RNA Pol II complex to assist in resolving R‐loops and mediating DNA damage repair, important in preventing oncogenesis [206]. This function of DHX9 is critical in maintaining appropriate cellular replication as expression of a dominant negative DHX9 protein fragment inhibited function of BRCA1 and led to defects in replication of mammary epithelial cells [207]. DHX9 truncation mutants have been identified in individuals with high risk of breast cancer, suggesting variants of DHX9 may promote oncogenesis [208]. DHX9 has also been implicated in the development of prostate cancer, and can have both oncogenic and tumor suppressor functions in lung cancer [209, 210, 211]. DDX5, in contrast, is generally described to have a consistently oncogenic effect through its regulation of the Wnt, Notch, mammalian target of rapamycin (mTOR), and forkhead box O3a (FOXO3a) pathways all involved in cancer progression [180, 212, 213, 214, 215]. As such, it has been identified as a pro‐cancer factor in lung cancer, colorectal cancer, and prostate cancer, among others [216, 217, 218]. Due to its well‐established oncogenic effects, a small molecule inhibitor of DDX5 called RX‐5902 has been developed that is currently undergoing phase two clinical trials [219]. Beyond these three helicases, several other DEAD/H‐box helicases have been shown to be involved in cancer, though these helicases are comparatively not as well studied in this context. These helicases include DDX1, DDX2, DDX6, DDX10, DDX20, DDX21, DHX32, DHX36, DDX41, DDX43, and DDX53 [179, 180]. Further study of the oncogenic mechanisms of these helicases may enhance our understanding of the mechanisms underlying the genesis and pathology of cancer.

### 5.3 Therapeutics Targeting DEAD/H‐Box Helicases

Most DEAD/H‐box helicases play crucial roles in infection, and, as a result, antiviral therapeutics targeting members of this family are in active development. For example, small molecule inhibitors of DDX2/eIF4A and DDX3, namely zotatifin and RK‐33, have been developed. Zotatifin is currently undergoing clinical trials, and RK‐33 has shown low toxicity and success in suppressing a broad range of viruses in vitro, including multiple strains of SARS‐CoV‐2 [31, 220, 221, 222]. Similarly, a DDX5 inhibitor is undergoing clinical trials, and due to DDX5's predominantly proviral role, this inhibitor may also prove valuable in treating viral infections [219]. In addition, several RIG‐I agonists are under investigation as potential antivirals or adjuvants for vaccines. Inarigivir, a dinucleotide‐derived small molecule agonist of RIG‐I, recently underwent Phase II clinical trials, showing efficacy in suppressing HBV infection, though hepatocellular dysfunction was also observed [223]. Two other RIG‐I agonists, KIN1148 and PUUC, have recently been used as adjuvants to augment immunity induced by IAV and SARS‐CoV‐2 vaccines and enhance protection from viral infection in murine models [224, 225]. To continue refining these existing therapeutics, and to develop new ones targeting other members of the DEAD/H‐box helicase family, many questions remain to be answered regarding the activity of these helicases. These helicases exhibit both proviral and antiviral roles, which appear to be regulated by temporal, cell‐type‐specific, and virus‐specific factors. Notably, some studies have reported contradictory results, with the same helicase having opposing effects in seemingly identical contexts. More careful investigation is needed to clarify the factors contributing to these discrepancies and to better understand the context‐specific mechanisms governing DEAD/H‐box helicase functions.

## 6 Conclusions and Future Directions

DEAD/H‐box helicases play a multitude of roles beyond their well‐known involvement in regulating viral infections and antiviral IFN responses. Although our understanding of these helicases has significantly advanced, much of the current knowledge stems from a limited number of studies in a few infection models. As recently noted, approximately 200 human and over 100 mouse proteins have been identified to contain the DEAD/H‐box domain [116]. As research continues to expand, it will be critical to explore the diverse mechanisms by which DEAD/H‐box helicases regulate immune responses across various diseases, cell types, and species. A more complete understanding of the regulatory landscape of these and other DEAD/H‐box helicases will help refine these existing strategies and reveal new avenues for the development of therapeutics targeting this critical family of helicases.

## Conflicts of Interest

The authors declare no conflicts of interest.

## Data Availability

The authors have nothing to report.
